# bsgenova: an accurate, robust, and fast genotype caller for bisulfite-sequencing data

**DOI:** 10.1186/s12859-024-05821-7

**Published:** 2024-06-05

**Authors:** Yance Feng, Fei Gao

**Affiliations:** 1 Agricultural Genomics Institute at Shenzhen, Chinese Academy of Agricultural Sciences, Shenzhen, China; 2 HIM-BGI Omics Center, Zhejiang Cancer Hospital, Hangzhou Institute of Medicine (HIM), Chinese Academy of Sciences (CAS), Hangzhou, China

**Keywords:** Genotype, SNP, BS-seq, Bisulfite-sequencing, DNA methylation

## Abstract

**Background:**

Bisulfite sequencing (BS-Seq) is a fundamental technique for characterizing DNA methylation profiles. Genotype calling from bisulfite-converted BS-Seq data allows allele-specific methylation analysis and the concurrent exploration of genetic and epigenetic profiles. Despite various methods have been proposed, single nucleotide polymorphisms (SNPs) calling from BS-Seq data, particularly for SNPs on chromosome X and in the presence of contaminative data, poses ongoing challenges.

**Results:**

We introduce bsgenova, a novel SNP caller tailored for bisulfite sequencing data, employing a Bayesian multinomial model. The performance of bsgenova is assessed by comparing SNPs called from real-world BS-Seq data with those from corresponding whole-genome sequencing (WGS) data across three human cell lines. bsgenova is both sensitive and precise, especially for chromosome X, compared with three existing methods. Moreover, in the presence of low-quality reads, bsgenova outperforms other methods notably. In addition, bsgenova is meticulously implemented, leveraging matrix imputation and multi-process parallelization. Compared to existing methods, bsgenova stands out for its speed and efficiency in memory and disk usage. Furthermore, bsgenova integrates bsextractor, a methylation extractor, enhancing its flexibility and expanding its utility.

**Conclusions:**

We introduce bsgenova for SNP calling from bisulfite-sequencing data. The source code is available at https://github.com/hippo-yf/bsgenova under license GPL-3.0.

**Supplementary Information:**

The online version contains supplementary material available at 10.1186/s12859-024-05821-7.

## Introduction

Bisulfite-conversion sequencing is an accurate and prevalently used technique to profile whole-genome DNA methylation (methylation at fifth position of cytosine, 5mC) at single-base resolution. Genotype or SNP calling from bisulfite-converted sequencing data, enabling allele-specific methylation analysis [1, 2] and joint analysis of genetic and epigenetic profiles such as quantitative trait loci analysis of DNA methylation (meQTL) [3, 4], is fundamental.

Compared with usual genotype calling from whole-genome sequencing (WGS) data, the task for BS-Seq data is faced with two primary obstacles. First, a part of cytosines (C) undergo transformation to thymine (T) in the sequenced data while the corresponding bases in the complementary strand transform from guanine (G) to adenine (A) during PCR amplification. But the exact transformed cytosines are unknown. Second, due to DNA templates destruction of bisulfite treatment, BS-Seq library quality is seriously affected by PCR duplicates and chimeric sequences among others. Possible base transformation and low-quality library make the read mapping for BS-Seq data more challenging than that for WGS data. As a result, genotype calling from BS-Seq data must tackle base conversion and low-quality mapping.

Bis-SNP [5], based on the Genome Analysis Toolkit (GATK) map-reduce framework, is the first formally published SNP caller from BS-Seq data that enables accurate SNP detection. At the same time, it is heavyweight and time-consuming. Latter MethylExtract [6], built on a simple model, is reported to be less specific but more sensitive compared to Bis-SNP. BS-SNPer [7], written in C, is fast and precision but not sensitive. Cgmaptools [8] is a toolkit dedicated for BS-Seq data analysis. For SNP calling, cgmaptools is sensitive but not robust against low-quality reads according to our evaluations. gemBS [9] is a thorough analysis pipeline adopted by ENCODE for BS-Seq data analysis including the functionality to correct methylation level from SNVs. However, its performance of whole-genome SNP calling has not evaluated for real-world BS-Seq data yet.

Despite the proposed methods for genotype/SNP calling from BS-Seq data in recent years, they fall short when compared to the well-developed counterparts for WGS data, such as bcftools [10], Mutect2 [11] from GATK4 toolkit, Strelka2 [12], DeepVariant [13], and others, in terms of performance and availability. Furthermore, the reported evaluations of published methods [5–9] for bisulfite-converted SNP calling are quite simple which ask for individual samples and fixed threshold or even simulated data.

**Fig. 1:**
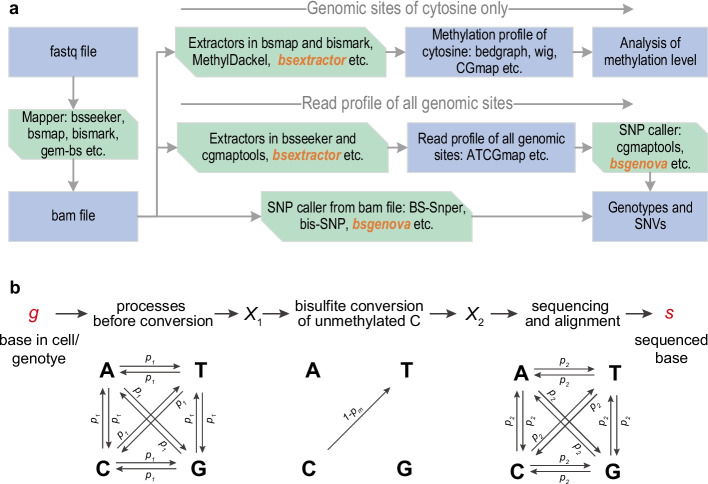
Workflow and model of bsgenova. **a** There are two ways to use bsgenova: two-step manner extracts an intermediate summary ATCGmap file of whole-genomic base coverages from bam file and then calls SNPs with ATCGmap file as input; one-step manner directly calls SNPs with bam file as input. bsgenova also integrates a coverage extractor bsextractor which can extract methylation information of cytosine sites and whole-genomic coverage as ATCGmap file. **b** A base in cell undergoes transforms during three modeled stages: processes before conversion, bisulfite conversion, and processes after conversion (sequencing and read alignment)

In our study of porcine oocyte methylome [14], we initially introduced the prototype of bsgenova along with basic evaluation, the allele-specific methylation analysis with the prototype given clear patterns and distinctions of different types of oocytes. To maximize the availability, recently, we reconstruct bsgenova with deep optimizations. The key improvements include: (1) bsgenova is reimplemented with Python and multi-process parallelization; (2) the likelihood and posterior computations of a batch of genome sites are optimized in matrix manipulation; (3) bsgenova maintains an in-memory cache pool from a stream of file and dispatches batches of data to each workhorse process; (4) bsgenova uses mapped bam file or an intermediate summary ATCGmap file as input; (5) to address the possible incompatibility of mapper and downstream SNP caller for WGBS data, we also implement bsextractor, a methylation extractor, which extracts methylation information as bedgraph and CGmap files and extracts read counts of all genome sites as ATCGmap file as illustrated by the workflow in Fig. 1a.

Benefited from these efforts, bsgenova is efficient in time, memory, and disk usages and flexible to cooperated with other tools. Most of all, the evaluations with WGBS and WGS data of three human cell lines from the Encyclopedia of DNA Elements (ENCODE) Project [15] affirm that bsgenova is accurate, as measured by precisions and sensitivities at different thresholds, and robust against contaminative library compared existing methods. For SNPs on chromosome X, the superiority of bsgenova is even more pronounced.

## Materials and methods

Bsgenova uses a summary ATCGmap file as input which includes the essential reference base, CG context, and ATCG read counts mapped onto Watson and Crick strands respectively. The output file includes a customized snv file and a conventional vcf file. The snv file records the posterior probabilities of 10 possible genotypes, and the posterior probability that this site is not a SNP (*p*-value), the posterior probability of homozygous genotype, and the derived posterior frequencies of A/T/C/G alleles. The vcf file records similar information in the specified format.

### Bayesian probabilistic model of bsgenova

Bsgenova builds a Bayesian probabilistic model of read counts for each specific genomic position to calculate the (posterior) probability of a SNP. We first formulate the probabilistic model for reads mapped to Watson strand only in a haploid genome. Then it is easy to extend model to the case of both Watson and Crick strands in a diploid genome on the top of simple-case model.

bsgenova makes the following assumptions,

1. Cytosine is methylated with probability ${p}_{m}$ depending on its sequence context CG (${p}_{m}^{(\text{CG})}$)or non-CG (${p}_{m}^{(\text{nonCG})}$);
2. Before bisulfite conversion, each base turns into other three bases with equal probability ${p}_{1}$ during sample preparation, ${p}_{1}$ models the errors of DNA replication, spontaneous deamination of methylated cytosines and others;
3. Bisulfite conversion is perfect, since the goal is to infer SNP not methylation, it is equivalent to regard all converted cytosines as “unmethylated” and all unconverted cytosines as “methylated” in the model;
4. After bisulfite conversion, each base turns into other three base with equal probability ${p}_{2}$ due to mistakes during PCR, sequencing, and read alignment;
5. DNA templates from Watson and Crick strands degrade uniformly, PCR is linear, and each DNA fragment is sequenced with equal probability;
6. The read counts mapped onto Watson strand indicating A, T, C, and G at specific site follow multinomial distributions, namely, ${\varvec{W}}=\left({A}_{\text{W}},{T}_{\text{W}},{C}_{\text{W}},{G}_{\text{W}}\right),{\varvec{W}}|g \sim \text{ multinomial}\left(n,{{\varvec{\mu}}}_{g}\right)$, $g\in \mathcal{G}=\{\text{A},\text{T},\text{C},\text{G}\}$ is the genotype, $n={A}_{\text{W}}+{T}_{\text{W}}+{C}_{\text{W}}+{G}_{\text{W}}$, ${{\varvec{\mu}}}_{g}$ is the vector of probabilities of each base in the reads with actual genotype $g$ provided.

According to the processes illustrated in Fig. 1b, each element of ${{\varvec{\mu}}}_{g}$ is the conditional probability of sequencing base $s$ with genotype $g$ provided,

$$P\left(s|g\right)=\sum_{{X}_{1}\in \mathcal{G}}\sum_{{X}_{2}\in \mathcal{G}}P(s|{X}_{2},g)P({X}_{2}|{X}_{1},g)P({X}_{1}|g), s, g\in \mathcal{G},$$

where ${X}_{1},{X}_{2}$ are the bases after the process of mutation and bisulfite conversion, respectively. The calculation is straightforward, for example $P\left(s=T|g=A\right)=2{p}_{1}-{p}_{1}{p}_{m}+{p}_{2}-4{p}_{1}{p}_{2}+2{p}_{1}{p}_{2}{p}_{m}$. The concise vector representation ${{\varvec{p}}}_{\text{g}}=\left(P\left(\text{A}|g\right),P\left(\text{T}|g\right),P\left(\text{C}|g\right),P\left(\text{G}|g\right)\right), g\in \mathcal{G}$ is useful in the extension of the model.

The prior distribution of $g$ is specified depending on the base $r$ reference genome and the parameter of mutation rate ${p}_{u}$,

$$P\left(g|r\right)=\left\{\begin{array}{cc}1-3{p}_{u},& g=r\\ {p}_{u},& g\ne r\end{array}\right..$$

The prior is the guess without any sequenced reads. After counting the reads, according to Bayes’ formula, the posterior probability of $g$ is given by

$$P\left(g|{\varvec{W}},r\right)\propto P\left({\varvec{W}}|g\right)P\left(g|r\right).$$

Now we turn to the counts of both Watson and Crick strands in a diploid genome, there are four major differences: (1) now there are 10, instead of 4, possible genotypes in diploid genome, i.e., $g\in \mathfrak{G}=\{\text{AA},\text{TT},\text{CC},\text{GG},\text{ AC},\text{AG},\text{AT},\text{CG},\text{CT},\text{GT}\}$; (2) the prior distribution should be updated on extended genotype space $\mathfrak{G}$ accordingly; (3) we assume the joint distribution of reads mapped to Watson and Crick strands as multinomial, i.e., ${\varvec{Z}}=\left({\varvec{W}},{\varvec{C}}\right)$ and ${\varvec{Z}}|g \sim \text{ multinomial}\left(n,{{\varvec{\mu}}}_{g}\right), g\in \mathfrak{G}$, where ${\varvec{W}}=\left({A}_{\text{W}},{T}_{\text{W}},{C}_{\text{W}},{G}_{\text{W}}\right)$ and ${\varvec{C}}=\left({A}_{\text{C}},{T}_{\text{C}},{C}_{\text{C}},{G}_{\text{C}}\right)$ are numbers of reads indicating A, T, C, and G mapped onto Watson and Crick strands, respectively, at the specific genomic position; $n$ is the total reads, $n={A}_{\text{W}}+{T}_{\text{W}}+{C}_{\text{W}}+{G}_{\text{W}}+{A}_{\text{C}}+{T}_{\text{C}}+{C}_{\text{C}}+{G}_{\text{C}}$, then the posterior probability of genotype is given by $P\left(g|{\varvec{Z}},r\right)\propto P\left({\varvec{Z}}|g\right)P\left(g|r\right)$; (4) the expected proportion of each sort of reads ${{\varvec{\mu}}}_{g},g\in \mathfrak{G}$ now is a vector of 8-dimension, according to the results of simple-case model, we have ${{\varvec{\mu}}}_{g}={{\varvec{\mu}}}_{ab}=\frac{1}{4}\left({{\varvec{p}}}_{a}+{{\varvec{p}}}_{b}, {{\varvec{p}}}_{\overline{a} }+{{\varvec{p}}}_{\overline{b} }\right), a,b\in \mathcal{G}=\{\text{A},\text{T},\text{C},\text{G}\}$ and $\overline{a }$ is the complement of $a$, i.e., $\overline{\text{A} }=\text{T},\overline{\text{T} }=\text{A},\overline{\text{C} }=\text{G}$, and $\overline{\text{G} }=\text{C}$.

The posterior $P\left(g|{\varvec{Z}},r\right)$, a vector of dimension 10, indicates the probability of each possible genotype in $\mathfrak{G}$. According to it, the “*p*-value” of a SNP is defined as the posterior probability of reference genotype $P\left(g=r|{\varvec{Z}},r\right)$; the posterior probability of a homozygote is $P\left(g\in \{\text{AA},\text{TT},\text{CC},\text{GG}\}|{\varvec{Z}},r\right)$; and the A, T, C, and G allele frequencies are estimated by the weighted means of posterior probabilities. These informative metrics are output by bsgenova.

### Likelihood and posterior probability by matrix computation

The likelihood $P\left({\varvec{Z}}|g\right)$ and posterior probabilities $P\left(g|{\varvec{Z}},r\right)$ of multiple genomic positions are easy to calculate via matrix multiplication. Considering the probability of 8-dimension multinomial distribution, ${\varvec{Z}}|g \sim \text{ multinomial}\left(n,{{\varvec{\mu}}}_{g}\right), g\in \mathfrak{G}$,

$$P\left({\varvec{Z}}={\varvec{z}}|g\right)=\frac{n!}{{\prod }_{k=1}^{8}z\left(k\right)!}\prod_{k=1}^{8}{\mu }_{g}{\left(k\right)}^{z\left(k\right)},$$

where $z\left(k\right)$ and ${\mu }_{g}\left(k\right)$ are $k$-th elements of ${\varvec{z}}$ and ${{\varvec{\mu}}}_{g}$, respectively. The log-likelihood

$$l\left({{\varvec{\mu}}}_{g};{\varvec{Z}}\right)=\log P \left({\varvec{Z}}={\varvec{z}}|g\right)=\sum_{k=1}^{8}z\left(k\right)\log {\mu }_{g}\left(k\right)+\text{constant}.$$

Suppose the reference bases of these positions are the same, then the unnormalized posterior probabilities of $N$ genomic positions are

$${\mathbf{P}}_{N\times 10}=\mathbf{P}\left(i,g\right)={\left(P\left({\varvec{W}}|g\right)P\left(g|r\right)\right)}_{i,g}=\text{exp}\left(\mathbf{Z}{\mathbf{M}}^{\text{T}}\right)\mathbf{D},$$

$${\mathbf{Z}}_{N\times 8}=\left(\begin{array}{c}{{\varvec{z}}}_{1}\\ \vdots \\ {{\varvec{z}}}_{N}\end{array}\right),$$

$${\mathbf{M}}_{10\times 8}=\left(\begin{array}{c}\log {{\varvec{\mu}}}_{g}^{\left(1\right)}\\ \vdots \\ \log {{\varvec{\mu}}}_{g}^{\left(N\right)}\end{array}\right),$$

$${\mathbf{D}}_{10\times 10}=\text{diag}\left(P\left(g|r\right), g\in \mathfrak{G}\right),$$

where ${{\varvec{z}}}_{i}$ is vector of reads at position-$i$ and ${{\varvec{\mu}}}_{g}^{\left(i\right)}$ is the vector of expected proportions of each kind of reads for genotype $g$, exp is the element-wise exponential. At last, each genomic position (row of $\mathbf{P}$) is normalized to sum to one.

## Results

### Real-world data and methods involved in the evaluation

Since Reduced Representation Bisulfite Sequencing (RRBS) only captures a small fraction of genome sites and shotgun Whole Genome Bisulfite Sequencing (WGBS) can capture most sites, to evaluate the performance of bsgenova, we compared the SNPs called from WGBS samples and SNPs called from WGS samples of the same cell line. All WGBS and WGS samples were downloaded from ENCODE (Table S1). In total, there were 8 WGBS samples (clean data of median ~ 79G bases or ~ 25 × depth) and 9 WGS samples (clean data of ~ 102 G bases or ~ 32 × depth) of three human cell lines A549 [16, 17], GM23248 [16, 18], and K562 [16, 17, 19]. Except bsgenova, we also carried out the same evaluations for three common methods including BS-Snper and two methods from cgmaptools (including cgmaptools_bayes and cgmaptools_binom) for comparison. All the evaluations were performed in a computer model with Intel Xeon E5-2620 v2 CPU, 250 GB DDR3 1600 MHz memory, and Centos with Linux kernel 3.10.

For each cell line each pair of WGBS sample and WGS sample was subjected to comparison. WGS samples were mapped to UCSC hg38 genome with bwa [20, 21], SNPs identified by both Mutect2 and bcftools were considered as true background. To mitigate the impact of coverage disparities between WGBS and WGS samples, only callable genomic sites, which were covered by at least 10 reads in two types of samples, were included in the evaluation. After depth filtering, the median genomic sites used for comparison was 2.58 Gbp (83.2% out of 3.1 Gbp, the size of human reference genome GRCh38). For each pair of samples, the callable set was unique. Furthermore, reads with map qualities less than 20 were excluded for all methods, the remaining reads were referred to as clean data.

### bsgenova is precise, sensitive, and resistant against contaminations

**Fig. 2:**
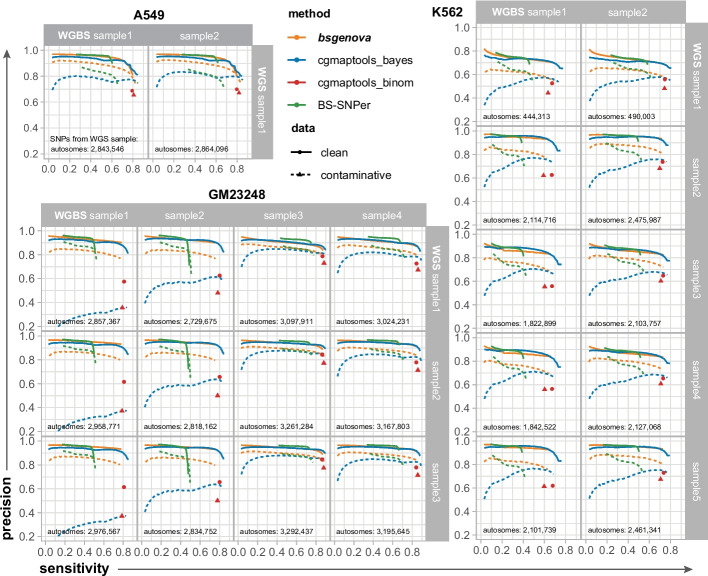
Evaluations of bsgenova and other methods calling SNPs on autosomes with real-world data. For each cell line and each pair of WGBS and WGS samples, the SNP intersection of Mutect2 (in GATK toolkit) and bcftools were regarded as truths, ROC of sensitivity and precision was calculated by tuning the threshold of *p*-value/score. cgmaptools_binom did not output a continuous score or *p*-value and hence manifested as a point. Only the callable genomic sites, namely covered by at least 10 reads in both WGBS and WGS samples, were included in the evaluation. The number of true SNPs of callable sites (in autosomes) was shown at the bottom. The clean data included the reads with map quality ≥ 20 (solid lines), while the contaminative data (dashed lines) included all the mapped reads without filtering low-quality reads to reproduce the intrinsic contamination introduced during WGBS library construction and sequencing

As shown in Fig. 2, with each facet representing a pair of WGBS and WGS samples, depicted the ROC (receiver operating characteristic) curves (solid lines) of different methods by tunning *p*-value/score threshold compared with the true SNPs called from corresponding callable sites of WGS sample. The number of true SNPs (on autosomes) was shown at bottom. Notably, maximal precisions of bsgenova, cgmaptools_bayes, and BS-Snper in most pairs of samples exceeded 90% even 95%. However, BS-Snper exhibited a limited ability to detect true SNPs evidenced by sharp drops of corresponding ROC curves at sensitivities between ~ 40% and 60%. In other words, BS-Snper may miss 40% ~ 60% SNPs regardless of threshold relaxation. bsgenova and cgmaptools_bayes exhibited greater sensitivity, detecting over 90% of the SNPs in most cases.

Given the prevalence of contamination and noise-induced low-quality omics data, especially for bisulfite-treated libraries, the robustness of data analysis models becomes crucial. Approximately 10% (Figure S1) of reads with map quality less than 20 were excluded from SNP calling in WGBS samples during the evaluations mentioned earlier. To assess the resistance of different methods against these intrinsic contaminations, we provided all mapped reads as input to each SNP caller keeping other processes constant. No additional in silicon artificial reads were introduced indicating the realism of the scenarios.

The parallel results for contaminative data were illustrated by the dashed lines alongside in Fig. 2. bsgenova exhibited limited declines for each sample of each cell line (indicated by the difference of solid and dashed lines) and maintained precisions greater than 80% in most pairs of samples while holding the same sensitivity. Conversely, cgmaptools exhibited a pronounced decline when dealing with unidealized data, with the precision dropping below 50% even below 20%, suggesting the underlying model collapsed due to the contaminations. At last, the performance reduction of BS-Snper was moderate.

### Performance of SNP calling on chromosome X

**Fig. 3:**
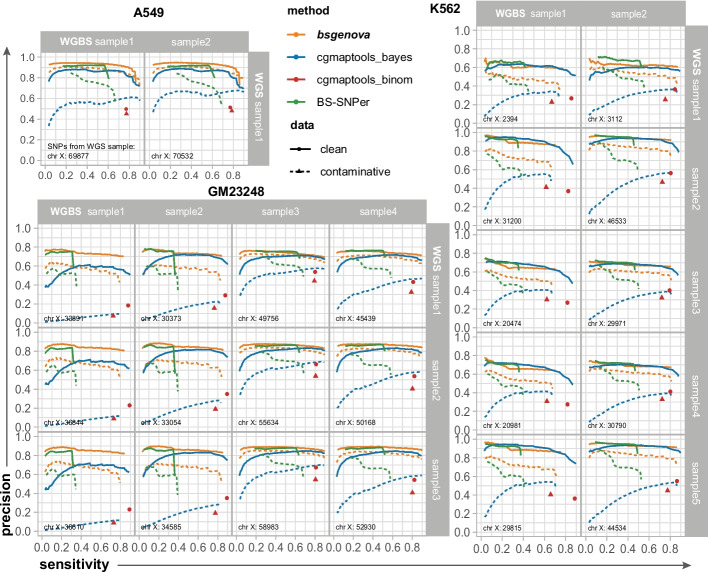
Evaluations of bsgenova and other methods calling SNPs on chromosome X with real-world data. The configurations are the same with Fig. 2 except that the SNPs under evaluation are on chromosome X. For male cell lines (A549 and GM23248), calling SNPs on chromosome X from WGBS data is harder. In this case, bsgenova outperformed other methods for both clean and contaminative data

Due to sequence homology and/or a reduced DNA content especially in male genome (A549 and GM23248 cell lines), SNP calling for chromosome X poses a greater challenge compared with autosomes. In Fig. 3, the SNP calling performance (ROCs) on chromosome X with the same processes as described above for different methods was illustrated. For cell lines A549 and GM23248, bsgenova outperformed other methods at various levels of sensitivity. Notably, when calling SNPs with contaminated data (depicted by dashed lines), the advantage of bsgenova became even more pronounced. These results suggest that bsgenova is well-equipped to handle challenging scenarios such as those for chromosome X or involving contaminative data, whereas existing methods, especially cgmaptools, exhibited subpar performance.

### Maximizing a linear combination of sensitivity and precision

To comprehensively compare the performance of different methods independent of thresholds, we consider a scalarization of sensitivity and precision instead of the common measure AUC (area under curve). This method is adopted because complete ROC was hard to calculate and in certain cases, sensitivity and precision may be discriminated with different degrees of importance. Specifically, we define the follow maximization,

$$M = \max \left\{ {\left( {1 - \alpha } \right) \times {\text{sensitivity}} + \alpha \times {\text{precision}}} \right\}{\text{, for fixed }} \alpha \in \left[ {0,1} \right].$$

When $\alpha =1/2$, sensitivity and precision are equally important; when $\alpha =5/6$, the importance of precision is five times that of precision indicating a fair tradeoff of a 5% decrease of sensitivity for a 1% increase of precision.

**Fig. 4:**
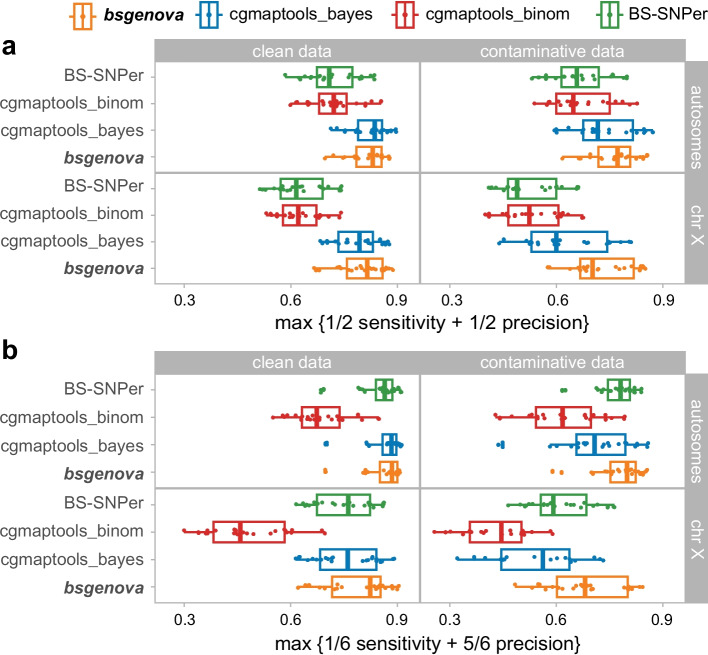
Maximizing linear combinations of sensitivity and precision. **a** In cases that sensitivity and precision are of equal importance, we set equal coefficients in the scalarization. **b** In cases that false positive calls are to be avoided preferentially, we set the coefficient of precision larger than that of sensitivity, say five times, which indicates a fair tradeoff of a 5% decrease of sensitivity for a 1% increase of precision

For $\alpha =1/2$ (Fig. 4a), both bsgenova and cgmaptools_bayes exhibited superior performance compared with BS-Snper and cgmaptools_binom for samples containing clean reads only (map quality $\ge 20$), in both autosomes and chromosome X. On the other hand, when setting $\alpha =5/6$ to reduce false positive SNPs (Fig. 4b), the comprehensive $M$ scores of BS-Snper increased across all cases. Notably, bsgenova outperformed the other methods particularly in the more challenging scenarios containing contaminations and for chromosome X.

### bsgenova is fast and resource-efficient

**Fig. 5:**
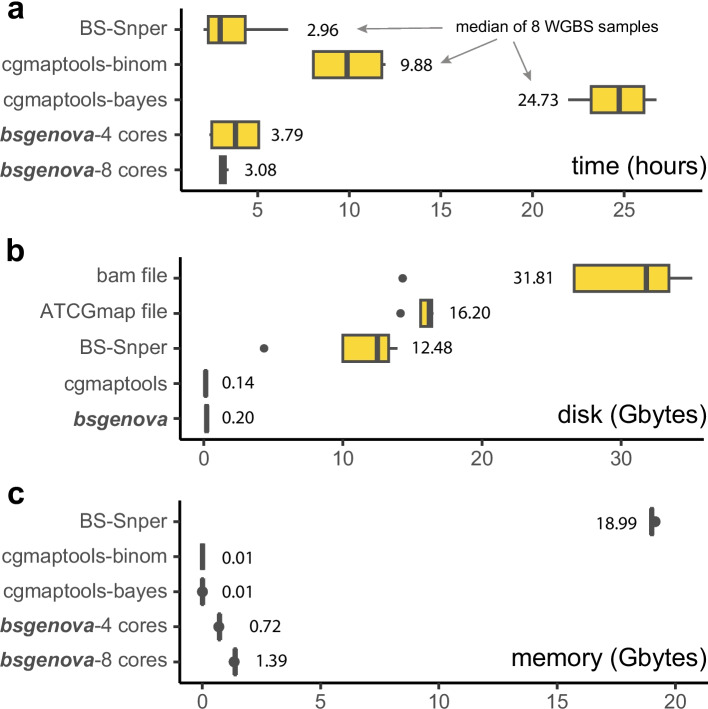
Resource usages of bsgenova and other methods. **a** bsgenova is implemented with multi-process parallelization consuming ~ 3 h for a WGBS sample of ~ 79 G bases (~ 25X depth) with 8 CPU cores. **b** cgmaptools must use an intermediate ATCGmap file as input, the disk usage of cgmaptools is nearly the same as that of BS-Snper. bsgenova has two ways to run: the same manner with cgmaptools (two-step) and use bam file as input which does not produce any temporary files (one-step). **c** bsgenova costs ~ 1 GB memory which is affordable in a modern computer

In addition to utilizing matrix computation, bsgenova incorporates multi-process parallelization for acceleration. bsgenova reads data from file or pipe and maintains an in-memory cache pool of data batches of genome intervals. The batches are then dispatched to workhorse processes. The synergy of matrix computation, parallelization, and caching rendered bsgenova both fast (~ 3 h for a sample of ~ 79G bases) and resource-efficient in terms of memory and disk usage (Fig. 5).

Summarizing these assessments, as indicated in Figure S2, BS-Snper exhibits limited sensitivity, identifying only ~ 40% to ~ 60% of all true SNPs, and is memory-intensive. cgmaptools is susceptible to contaminative/unideal data and is time-consuming. In contrast, bsgenova strikes a balance in these evaluated aspects, making it a versatile tool.

### Read depth and parameter tuning

To assess the impact of read depth to bsgenova, we grouped all genomic sites by their read depths in WGBS sample and calculated the precision and sensitivity of bsgenova on the grouped sites of certain read depth, respectively (Figure S3). Generally speaking, with fixed *p*-value threshold, the sensitivity increases as the depth increases while the best read-depth maximizing precision is approximately 15–20.

bsgenova accepts parameters such as mutation rate, error rate, and cytosine methylation rates in both CpG and non-CpG contexts. to assess the dependence of bsgenova on these parameters, we compared the differences of SNP outputs with various parameter combinations (Figure S4). bsgenova reported consistent results with different tuning parameters which suggested its robustness against model (parameters) misspecification.

## Discussion

In this report we introduced bsgenova as a novel SNP caller for bisulfite-converted sequenced data such as WGBS and RRBS data. To make bsgenova performant and readily available we paid many efforts to the optimizations of probabilistic model and implementation as shown the results of evaluation and a summary table (Figure S2).

Up to now, the published methods to the best of our knowledge, including bsgenova, only model the genotypes of homozygote and heterozygote of a diploidy genome, namely the germline single-nucleotide variation (SNV) with respect to reference genome. The mutation of short indel is hard to call by our experience due to low quality of WGBS library. On the other hand, somatic SNV of a small fraction of cells can neither be called from WGBS library of tissue sample such tumors. Limited by the destruction and conversion of bisulfite as well as the statistical models used, mutation calling from bisulfite-converted data can only report confidant results for germline SNVs.

An advance of this issue is to use harpin prime during the library construction to link two strands of a DNA fragment in a single read in the sequenced data. Linking two strands makes sure that both Watson and Crick strands are sequenced at the same time which facilitates SNP calling from bisulfite-converted library. Liang et al. reported a significant improvement of SNP calling utilizing harpin bisulfite sequencing [22]. However, additional protocol steps with harpin prime reduce the DNA template utilization rate, hence this method is not available in situations with low DNA content input such as at single-cell level. In addition, due to the linkage of two strands, harpin bisulfite sequencing makes double-ended sequencing to degenerate to single-ended sequencing substantially.

SNP calling from bisulfite-converted data to enable joint analysis of genetic and epigenetic information remains challenging. Improvements of both library construction and corresponding robust probabilistic models need to be made to detect both DNA methylation and genotype accurately at whole-genome scope.

### Supplementary Information

Additional file1 

## Data Availability

All analyses were done on publicly available data from ENCODE (Table S1). The source code of bsgenova and bsextractor is available at https://github.com/hippo-yf/bsgenova.
