# Supplementary material for: bsgenova: an accurate, robust, and fast genotype caller for bisulfite-sequencing data
Source: BMC Bioinformatics. 2024 Jun 5;25:206. doi: 10.1186/s12859-024-05821-7 (PMC11151569; doi:10.1186/s12859-024-05821-7)
Supplement: Supplementary file 1 — Additional file1 [file 12859_2024_5821_MOESM1_ESM.docx]

| **Table S1. WGBS and WGS samples of three human cell lines from ENCODE.** | | | | |
| --- | --- | --- | --- | --- |
| **Sample** | **Clean Data (GBases)** | **ENCODE accession** | **Cell Line** | **Library** |
| sample1 | 91.2 | ENCSR481JIW | A549 | WGBS |
| sample2 | 93.3 | ENCSR481JIW | A549 | WGBS |
| sample1 | 73.4 | ENCSR440MLE | GM23248 | WGBS |
| sample2 | 63.3 | ENCSR440MLE | GM23248 | WGBS |
| sample3 | 101.0 | ENCSR625HZA | GM23248 | WGBS |
| sample4 | 84.1 | ENCSR625HZA | GM23248 | WGBS |
| sample1 | 51.3 | ENCSR765JPC | K562 | WGBS |
| sample2 | 54.1 | ENCSR765JPC | K562 | WGBS |
| sample1 | 103.8 | ENCSR521ELB | A549 | WGS |
| sample1 | 152.8 | ENCSR016AIX | GM23248 | WGS |
| sample2 | 101.7 | ENCSR674PQI | GM23248 | WGS |
| sample3 | 107.7 | ENCSR674PQI | GM23248 | WGS |
| sample1 | 22.2 | ENCSR025GPQ | K562 | WGS |
| sample2 | 101.9 | ENCSR045NDZ | K562 | WGS |
| sample3 | 93.5 | ENCSR053AXS | K562 | WGS |
| sample4 | 94.6 | ENCSR053AXS | K562 | WGS |
| sample5 | 246.1 | ENCSR711UNY | K562 | WGS |

| 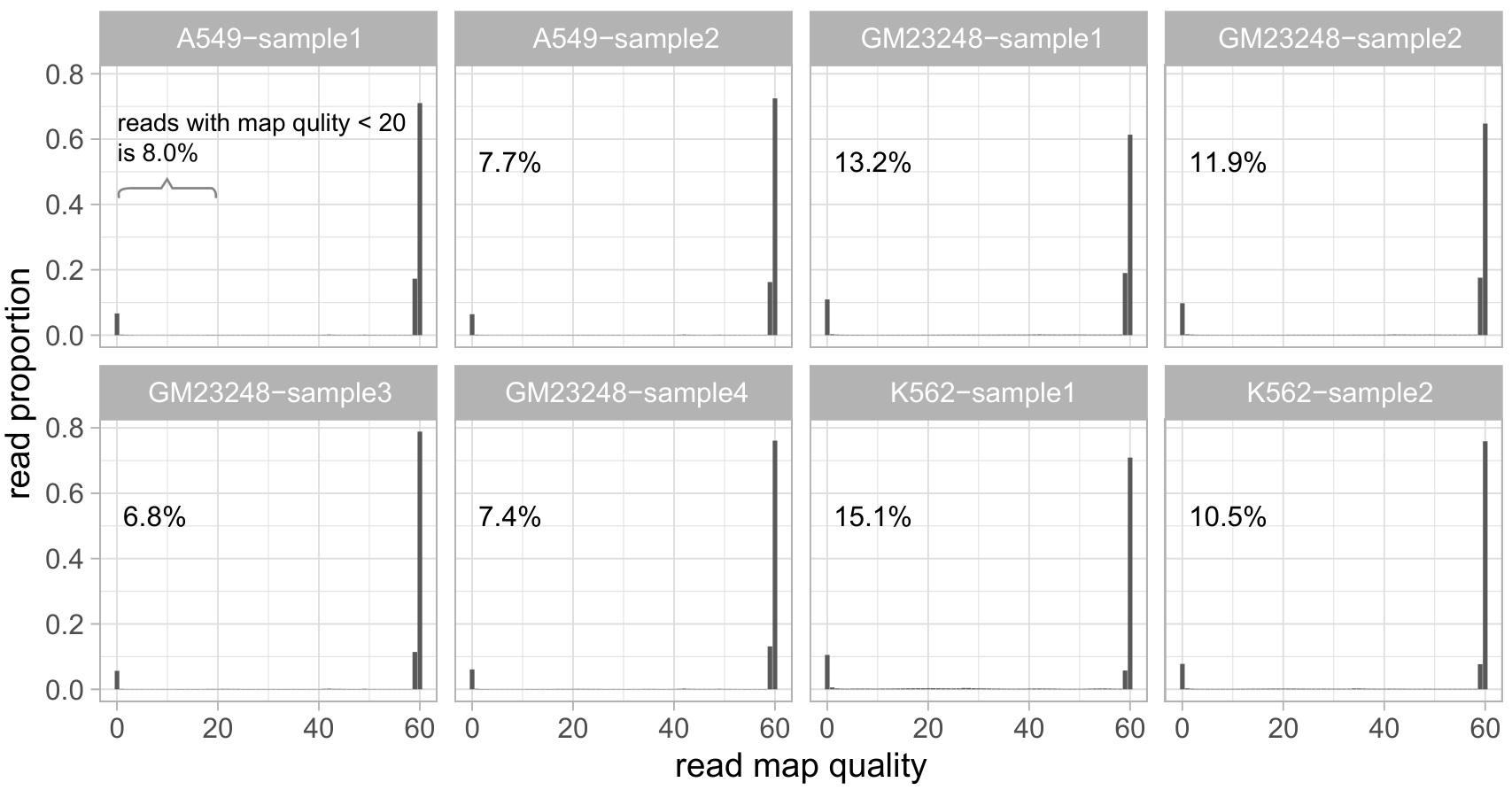 | |
| --- | --- |
|  |  |
| **Figure S1. Distributions of read map qualities for each WGBS sample.** The proportion of intrinsic low-quality reads (quality< 20) is approximately 10% after PCR deduplication. |  |

| 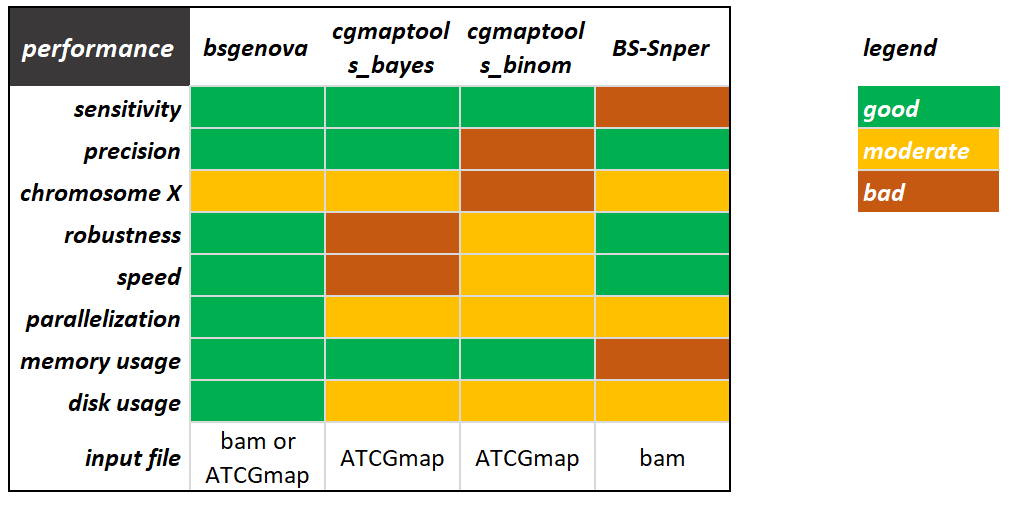 |
| --- |
| **Figure S2. Performance summary of bsgenova and other existing three methods from two packages, cgmaptools and BS-Snper.** bsgenova is balanced in each aspect compared with others. |

| 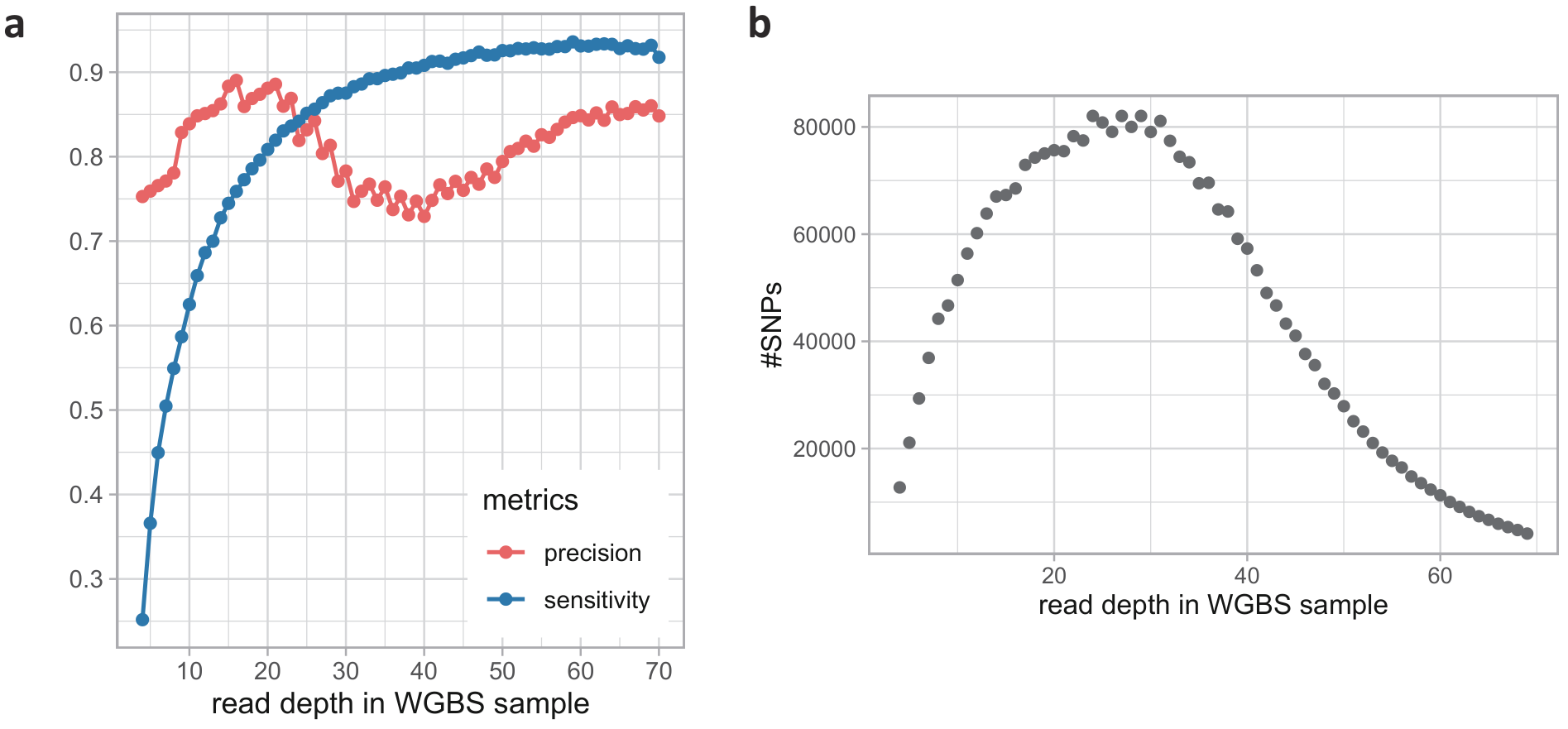 |
| --- |
| **Figure S3. The performance of bsgenova of genomic sites with various read depth.** For WGBS sample1 of cell line A549, bsgenova was used to call SNPs with *p*-value threshold 0.001. **(a)** For genomic sites with various read depth in WGBS sample (sites with depth = k instead of depth >= k, 4 <= depth <= 70), the sensitivity increases as the depth increases while the precision increases only for sites of less reads. The best read-depth maximizing precision ranges from 15 to 20. **(b)** The read depth distribution of SNPs called by bsgenova from WGBS sample1 of cell line A549. |


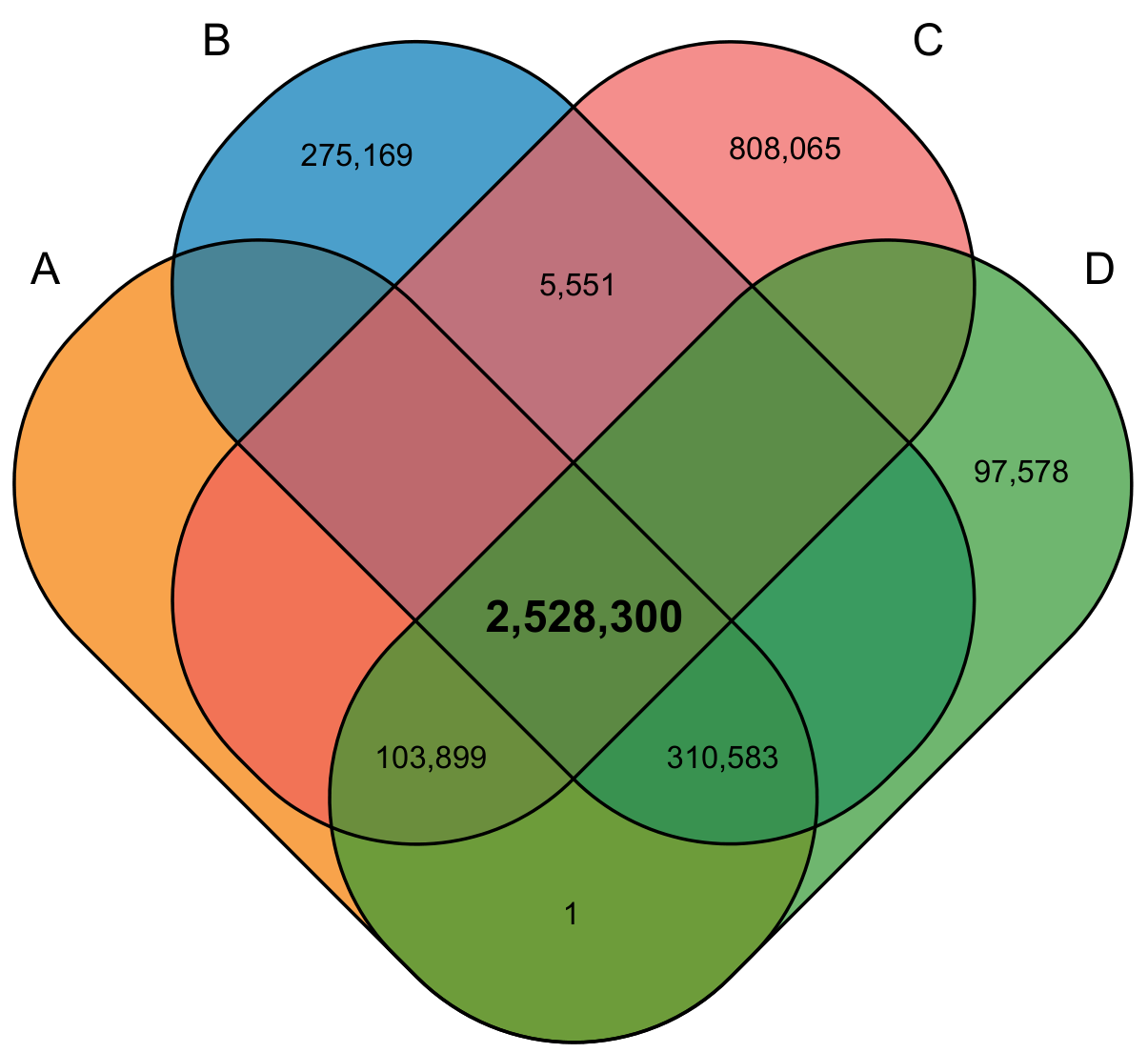


| **Parameters** | **mutation rate** | **depth threshold** | ***p*-value** | **error rate** | **CpG DNAme level** | **non-CpG DNAme level** | **number of SNPs** |
| --- | --- | --- | --- | --- | --- | --- | --- |
| A | 10^-3^ | 10 | 10^-3^ | 0.05 | 0.6 | 0.02 | 2,942,783 |
| B | 10^-3^ | 10 | 10^-3^ | 0.01 | 0.6 | 0.02 | 3,119,603 |
| C | 10^-3^ | 10 | 10^-6^ | 0.05 | 0.8 | 0.05 | 3,445,815 |
| D | 10^-3^ | 8 | 10^-3^ | 0.05 | 0.6 | 0.02 | 3,040,361 |

**Figure S4. Compare the SNPs called by bsgenova with different parameters.** For the A549 WGBS sample1, we used different combinations of tuning parameters (A/B/C/D) of bsgenova as listed in the table to call whole-genome SNPs. bsgenova reported consistent results suggested by the intersection set of four outputs (2,528,300 SNPs).
